# Structural diversity of tick-borne encephalitis virus particles in the inactivated vaccine based on strain Sofjin

**DOI:** 10.1080/22221751.2023.2290833

**Published:** 2023-12-11

**Authors:** Andrey Moiseenko, Yichen Zhang, Mikhail F. Vorovitch, Alla L. Ivanova, Zheng Liu, Dmitry I. Osolodkin, Alexey M. Egorov, Aydar A. Ishmukhametov, Olga S. Sokolova

**Affiliations:** a Faculty of Biology, Lomonosov Moscow State University, Moscow, Russia; b Faculty of Biology, Shenzhen MSU-BIT University, Shenzhen, People’s Republic of China; c FSASI “Chumakov FSC R&D IBP RAS” (Institute of Poliomyelitis), Moscow, Russia; d Sechenov First Moscow State Medical University, Moscow, Russia; e Kobilka Institute of Innovative Drug Discovery, School of Medicine, Chinese University of Hong Kong, Shenzhen, People’s Republic of China; f Department of Chemistry, Lomonosov Moscow State University, Moscow, Russia

**Keywords:** Tick-borne encephalitis virus, inactivated vaccines, cryoelectron microscopy, image analysis, variability analysis, asymmetry

## Abstract

The main approach to preventing tick-borne encephalitis (TBE) is vaccination. Formaldehyde-inactivated TBE vaccines have a proven record of safety and efficiency but have never been characterized structurally with atomic resolution. We report a cryoelectron microscopy (cryo-EM) structure of the formaldehyde-inactivated TBE virus (TBEV) of Sofjin-Chumakov strain representing the Far-Eastern subtype. A 3.8 Å resolution reconstruction reveals the structural integrity of the envelope E proteins, specifically the E protein ectodomains. The comparative study shows a high structural similarity to the previously published structures of the TBEV European subtype strains Hypr and Kuutsalo-14. A fraction of inactivated virions exhibits asymmetric features including the deformations of the membrane profile. We propose that the heterogeneity is caused by inactivation and perform a local variability analysis on the small parts of the envelope protein shell to reveal membrane curvature features possibly induced by the inactivation. The results of this study will have implications for the design of novel vaccines against diseases caused by flaviviruses.

## Introduction

Tick-borne encephalitis (TBE) is one of the most hazardous vector-borne zoonotic infections in Northern Eurasia, with tens of thousands of clinical cases annually. Severe forms of the disease manifest as neurological syndromes, leading to a substantial deterioration of the quality of life, disability, or death. The infection is caused by the tick-borne encephalitis virus (*Orthoflavivirus encephalitidis,* TBEV), which may be transmitted through an infected tick bite or consumption of raw milk from infected mammals [1]. A lot of efforts have been made in the field of TBE drug discovery [2], but vaccination remains recommended by the World Health Organization (WHO) as the primary preventative measure against TBE. All commercially available TBE vaccines are based on the usage of inactivated TBEV as an antigen and have a very good safety and efficiency profile [1].

TBEV belongs to the tick-borne flavivirus group within the *Orthoflavivirus* genus (family *Flaviviridae*) [3]. Various TBEV subtypes have been identified, including the European subtype common in Europe and the Siberian and Far-Eastern subtypes, which impact most of Russia and may cause a more severe clinical manifestation [1]. Only the European subtype was studied in previous cryoelectron microscopy (cryo-EM) experiments [4,5]. TBEV is an enveloped RNA virus with a positive-sense single-stranded genome. It shares close structural similarities with other orthoflaviviruses, such as dengue, Zika, and West Nile viruses, according to cryo-EM studies at a near-atomic resolution [4,6–8].

The mature TBEV virions are nearly spherical particles (diameter approx. 50 nm) with icosahedral symmetry. The virion contains a nucleocapsid surrounded by a lipid membrane envelope and a shell of envelope protein E ectodomains. The nucleocapsid consists of core protein C and viral RNA, but the structure of the nucleocapsid has not been solved yet due to its asymmetric and/or disordered organization [4,5,9–11]. The lipid bilayer envelope anchors the transmembrane domains of 180 copies of the membrane-associated protein M and the envelope protein E. The membrane envelope is not perfectly spherical, following the icosahedral inner surface of the protein shell and the outer surface of the nucleocapsid [4,5]. Elongated ectodomains of the E protein are located at the outer surface of the lipid bilayer, forming a characteristic “herringbone” pattern [4,5], and serve as the antigens for most of the antibodies induced by TBEV [12,13]. The building block of the protein shell is a heterotetramer consisting of two E and two M proteins. Three neighboring heterotetramers form a tetragonal “raft” structure. The asymmetric unit, with respect to the overall icosahedral symmetry of the shell, corresponds to the half of the raft, i.e. three copies of E and three copies of M protein [5].

Although vaccines based on the inactivated TBEV virions are widely used in the clinical practice and show almost perfect protective efficacy [14], no high-resolution structural data are currently available for the chemically inactivated virions in the vaccine preparations. TBE vaccine manufacturing protocols are typically based on chemical inactivation of the virus by cross-linking agents, which may introduce substantial modifications to the virion structure [15]. Direct determination of the structure of the vaccine virions and its comparison with the structure of the native virions would help to assess the conservation of virion integrity and stability, as well as the antigenic epitope preservation after the inactivation [16].

In this study, we performed a cryo-electron microscopy visualization and structure reconstruction for the inactivated TBEV virions from a commercial TBE vaccine based on Sofjin-Chumakov strain. This is also the first study of the structure of a Far-Eastern subtype strain of TBEV. Structure reconstruction revealed a heterogeneity in the membrane envelope, which causes the inactivated virions to be not perfectly icosahedral. At the same time E proteins, which serve as antigenic epitopes, are well preserved on the surface of the particle.

## Materials and methods

### TBEV preparation

TBEV strain Sofjin-Chumakov (Genbank KC806252) is a prototype strain of the Far-Eastern subtype and used to produce commercial inactivated TBE vaccines (FSASI “Chumakov FSC R&D IBP RAS” (Institute of Poliomyelitis), Moscow, Russia) [17]. Reproduction, inactivation, and purification of TBEV was performed as described in [18, 19]. Virus was inactivated by 0.02% formaldehyde and stored in TNE/5 buffer solution (20 mM Tris-HCl, 25 mM NaCl, 1 mM EDTA, pH 7.8).

### Cryo electron microscopy

Cryo electron microscopy was performed in the Kobilka Cryo-Electron Microscopy Centre (KEMC) of Kobilka Institute of Innovative Drug Discovery, CUHK(SZ), Guangdong Province, China. 3 μL of the inactivated TBEV suspension were applied onto 200 mesh carbon on copper Quantifoil 1.2/1.3 grids, which were glow discharged with Tergeo Plasma Cleaner (PIE Scientific). The grids were vitrified using Vitrobot mark IV (ThermoFisher Scientific) with the blot time of 3 s at 100% humidity and 4°C temperature.

Data collection was performed with Titan Krios 300 kV cryo electron microscope (ThermoFisher Scientific), equipped with Gatan K3 direct detector in counting mode with a 20 eV energy filter slit width (Gatan, USA). A total of 3156 movies were acquired with the pixel size 0.85 Å, total dose 68 e/Å^2^ and average defocus of 1.5 μm.

### Data processing

Single particle data processing was carried out with cryoSPARC software [20]. After motion correction and CTF estimation the manually picked particles were used to create picking templates. After the template-based picking and initial round of 2D classification, a total of 73,553 particles were retained for further processing. To analyse the heterogeneity, specifically the asymmetric features of the membrane and nucleocapsid, several subsequent rounds of 2D classification were carried out. Those were performed with 50–100 classes, “initial classification uncertainty factor” of 10, and the tightly real-space windowed particles to exclude the strong density of the envelope proteins from the classification. After excluding the class averages with any asymmetrical features, a total of 8429 particles were kept. This subset will be referred to as the most symmetrical particles.

Ab-initio reconstruction of the icosahedral particles is prone to the artifacts, the flat and smeared density maps instead of the correct icosahedral shape. Instead of ab-initio reconstruction, the EMD-3752 map of the intact TBEV was used as an initial reference map. Homogeneous refinement with the icosahedral symmetry and 40 Å initial lowpass filter was followed with local CTF refinement and local motion correction. The reconstruction with the overall reported resolution of 3.6 Å was obtained at this step. The same processing was done on the subset of the most symmetrical particles, giving the map at the 3.8 Å resolution.

For both the full and most symmetrical particle subsets, icosahedral symmetry expansion was performed to examine the heterogeneity of individual rafts. Following the symmetry expansion, the particles were re-boxed to retain only one raft within the density map box. The rafts were positioned on the edges of the icosahedron, centered on the C2 axis, and spanning between two C5 axes. The new box was centered on the raft in a way that the raft C2 symmetry axis was collinear with the box Z axis. The homogeneous reconstruction of the reboxed particles was then used to generate the consensus reference map for subsequent processing.

It is important to highlight that in the subsequent processing we used the volume containing the entire raft, comprising two asymmetric subunits, as it provides sufficient mass for proper processing. During this stage, we strictly limited the angular and translational search to prevent the overlap of multiple copies of the same symmetry-expanded particle in the close orientations. This limitation is critical to avoid overfitting, as multiple identical overlapping projections can lead to inaccurate results.

Both full and the most symmetrical particle subsets were individually refined against the corresponding consensus reference maps. The mask encompassing all E and M proteins of the raft was generated from the protein models docked into consensus reference map in USCF Chimera [21]. The mask border was soft-padded with a width of 18 pixels. Subsequently, the local non-uniform masked asymmetric refinement was performed with a penalty imposed on rotations and shifts from the known pose and re-centering rotations and shifts at each interaction.

The particles with refined orientations were used for the 3D variability analysis employing three eigenvectors, no symmetry and a filter resolution of 4 Å. The resulting 3D variability plot was manually inspected and clustered into four clusters. Particles corresponding to each cluster were then subjected to the homogeneous reconstruction.

The atomic models for the E and M proteins were generated by the homology modelling based on the 5O6A template. The homology model was fitted as a rigid body into the locally sharpened density map using USCF Chimera. The set of the distance restraints within 5 Å around each atom was created in Coot [22]. The entire model then underwent real-space refinement in two steps, with the Geman Mclure alpha set to 0.01 and 0.1, respectively. The model of the asymmetric unit was further processed in ISOLDE [23] to optimize it against the density map. Subsequently, the model was refined with Phenix real space refinement [24] using the ISOLDE-processed model as a reference. The validation statistics were generated in Phenix to assess the quality and accuracy of the final atomic model. The local sharpening of the density map was also carried out in Phenix.

## Results

### Lack of perfect symmetry of the inactivated TBEV virions

**Figure 1.**
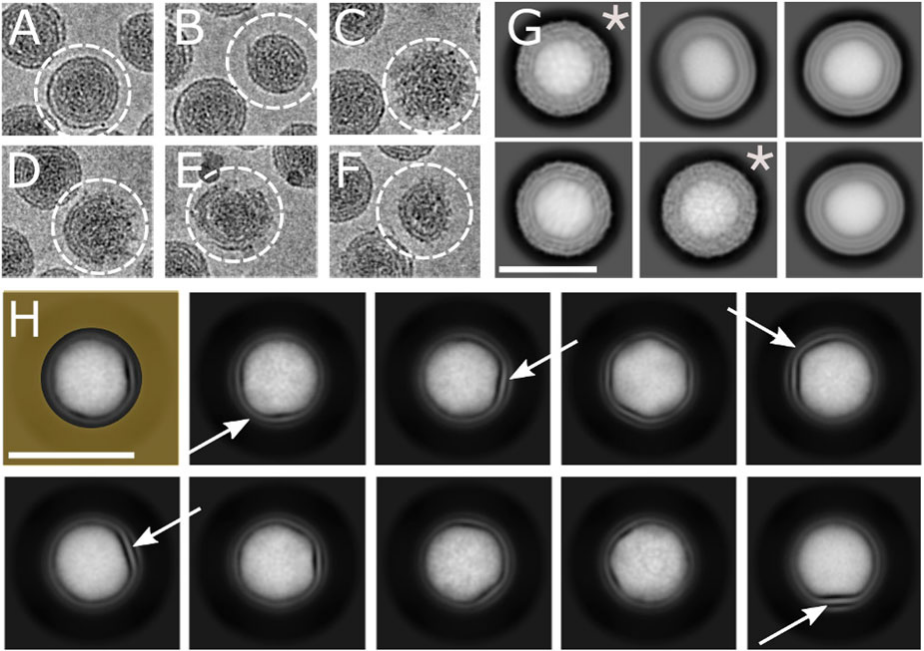
Two-dimensional classification of TBEV particles exhibits conformational heterogeneity of the dataset. (A–F) – Representative projections of inactivated TBEV particles: (A) – Round intact particle, (B) – Squeezed particle, (C) – Immature particle, (D) – Half-mature particle, (E, F) – Damaged particles; (G) – 2D class averages with marks indicating smooth and symmetric class averages, while others possess distorted, elongated shapes; (H) – Masked 2D classification reveals the asymmetric features of the membrane and the nucleocapsid. The circular mask includes only the central part of particle images and is shown overlapped with the first class average. Arrows indicate the asymmetric features.

The cryo-EM study was performed for the formaldehyde inactivated TBEV virions of the Sofjin-Chumakov strain, on which a commercial tick-borne encephalitis vaccine is based. The purified sample [18, 19] consisted of mostly spherical virions with an approximate diameter of 50 nm. Sample also contained a minute fraction of distorted particles, which were visually different from the normal particles. These distorted particles correspond to damaged, immature, and half-mature virions [25, 26] (Figure 1). By manually examining the dataset, we concluded that only 2% of the observed particles could be treated as distorted. Thus, the overall integrity and stability of the inactivated TBEV preparation after storing it at 4°C for several weeks was confirmed. The distorted particles were excluded from further single particle data processing.

Using single particle analysis, we separated the projections of the most symmetric and round virions from the elongated and severely damaged ones (Figure 1G,H). Classification focused on the central part of the virions revealed that about 20% of particles exhibit strong asymmetric features in both the membrane and nucleocapsid regions (Figure 1H). Subsequent rounds of 2D classification allowed us to select the subset composed of the most symmetric virion projections, where class averages do not exhibit any visible elongation. This symmetrical subset contained only 11% of the total number of all virion images.

### Molecular architecture of inactivated TBEV

The amino acid sequences of the proteins in the vaccine strain Sofjin-Chumakov [17] are quite similar to the ones of previously published TBEV European subtype structures, representing the intact HYPR strain (PDB ID 5O6A [4]) and the UV-inactivated Kuutsalo-14 strain (PDB ID 7Z51 [5]) (Supplementary Table 1).

**Figure 2.**
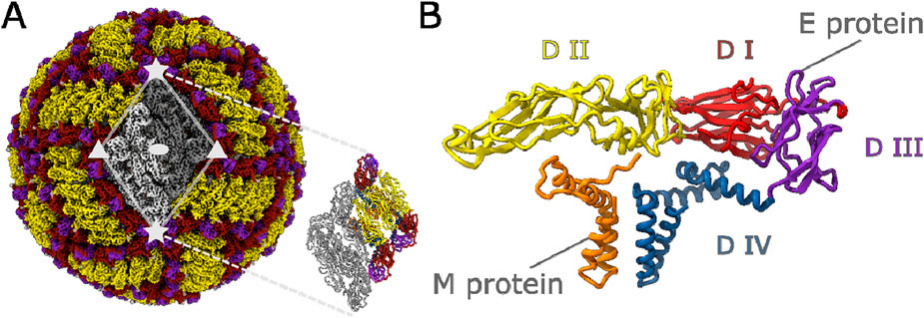
Overview of formaldehyde-inactivated TBEV structure. (A) – Isosurface representation colored according to the E protein domains. Two-fold, three-fold, and five-fold symmetry axes are marked with ellipse, triangle, and star respectively. The raft assembly consting of two asymmetric units is shown in the zoom panel. Asymmetric unit includes three E and three M protein chains and is represented by colored chains in the raft assembly. (B) – Cartoon representation of the E-M protein heterodimer colored by domain organization.

We performed a 3D reconstruction of the TBE vaccine virus particle using the cryo-EM single particle analysis (Figure 2). The overall composition of the virion closely resembles the previously published structures of native and UV-inactivated TBEV [4, 5]. The density map with the overall resolution of 3.8 Å allowed us to resolve the E and M proteins. The local resolution for the E protein ectodomain region reached 3.0 Å, which allowed a reliable model building. The envelope proteins were arranged into the icosahedral symmetrical “herringbone” pattern and could be grouped into “rafts” (Figure 2A). The outer surface of the virion is almost round with an approximate diameter of 50 nm. Each of the 60 asymmetric units consisted of three copies of E and three copies of M protein. Two neighboring asymmetric units formed the raft cornered by two C3 and two C5 rotational axes.

The resolution of the obtained density map was sufficient to build atomic models for the E and M proteins of the asymmetric unit. The domain composition of both proteins is shown in Figure 2B. The atomic models revealed that the structure of the inactivated virion is almost identical to the structure of intact virion of TBEV strain Hypr, PDB ID 5O6A [4]. It shows also a substantial similarity to the structure of UV inactivated TBEV strain Kuutsalo-14, PDB ID 7Z51 [5]. By aligning our E-M heterodimer to these reference structures, we found the overall E protein Cα RMSD to be 1.26 Å from Hypr and 1.22 Å from Kuutsalo-14. The M protein RMSD is much larger, 2.19 and 4.24 Å, respectively.

The local RMSD map shows a high degree of E protein ectodomain similarity with the structure of the intact TBEV strain Hypr (Supplementary Figure S1). Thus, we can confirm that the antibody epitopes on the surface of the virion should be well conserved after the inactivation with formaldehyde, as could be inferred from the efficacy of inactivated vaccines. The Asn154 glycosylation by N-acetyl-D-glucosamine is also retained. On the other hand, we did not observe any additional modifications introduced by formaldehyde, supporting the stochastic nature of the inactivation process.

Three E-M protein pairs within an asymmetric unit are considered to be quasi-equivalent, meaning that only subtle local differences may exist between the copies of the proteins within one asymmetric unit. Indeed, RMSD between superimposed E proteins of the asymmetric unit is in the range of 1.0–1.1 Å, while the M protein chains show an RMSD of 1.8–2.8 Å (Supplementary Figure S2). However, it should be noted that the deviations for the M protein may emerge from the low local resolution of the cryo-EM density map rather than from the actual structural features.

Minor displacements of the E protein domains III in our structure with respect to their location in the structure of the intact TBEV strain Hypr could be observed upon alignment of the full models of the asymmetric unit containing three dimers instead of the models of individual E-M heterodimers (Supplementary Figure S1). Given the quasi-equivalence of the heterodimers in the asymmetric unit, we suggest that these subtle differences do not represent any substantial structural changes, but may be attributed to the inaccuracies in the density map at the edges of the raft reconstruction. These inaccuracies arise from aligning a heterogeneous set of particles, each corresponding to a slightly different local structure.

The stem-anchor regions of envelope proteins differ significantly from the previously published structures, according to the local RMSD maps (Supplementary Figure S1). The deviations from the structure of the TBEV strain Hypr virion are most likely caused by the imprecision of the atomic model fitting. Our cryo-EM density map has a low resolution in the transmembrane region (>6 Å), which only allows the rigid body fitting of the alpha-helices. The deviations in comparison to the TBEV strain Kuutsalo-14 transmembrane region are more prominent, because Kuutsalo-14 M protein has an additional stabilizing interaction between the adjacent M subunits, caused by substitution of Arg40 with Lys40 [5].

### Structural variability of the transmembrane region

**Figure 3.**
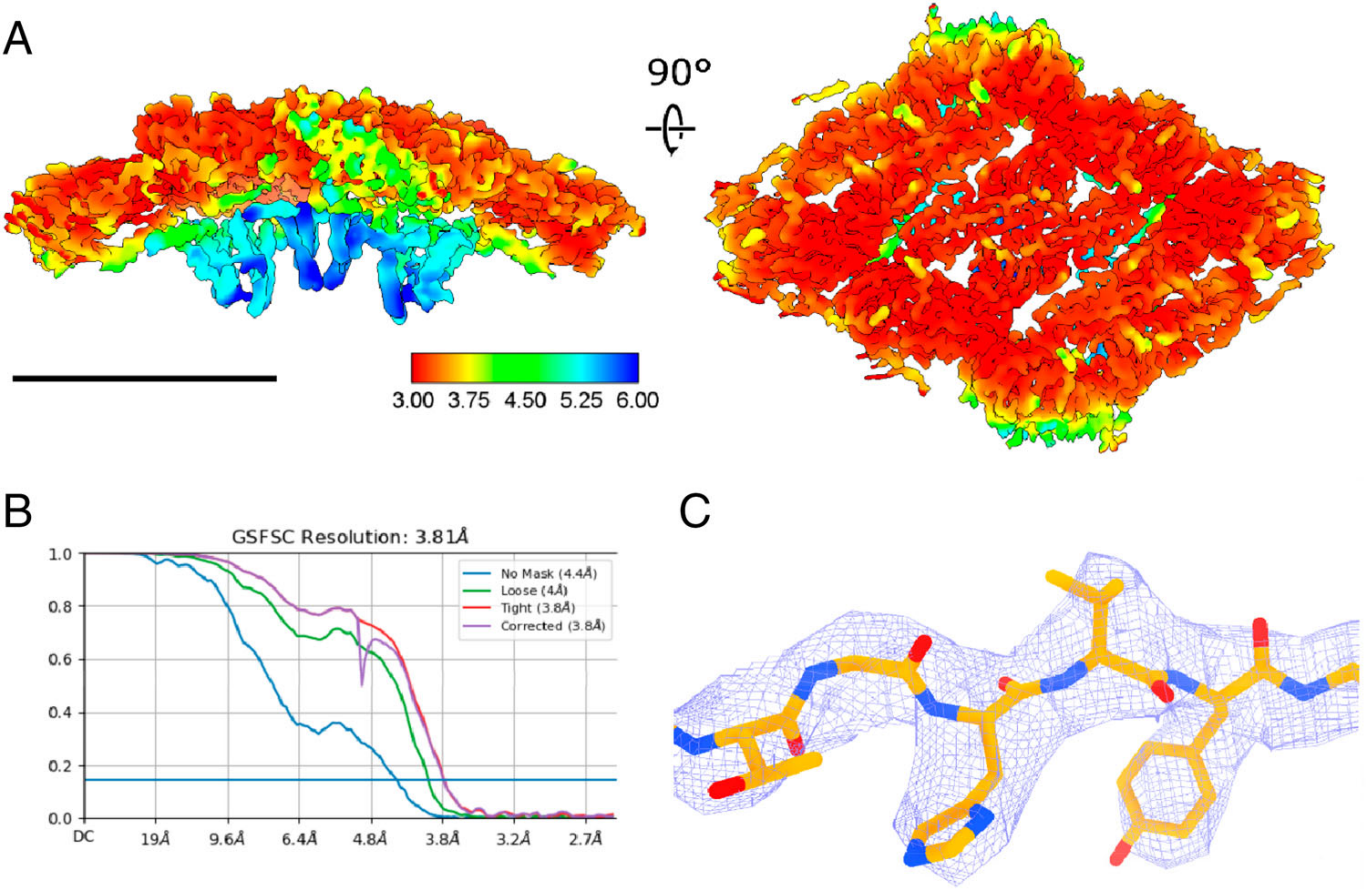
Local reconstruction of a single envelope protein raft (two asymmetric units). (A) – Locally sharpened density map, colored according to the local resolution, scale bar 10 nm, (B) – Global resolution estimation with Fourier shell correlation, (C) – Visualization of model fitting, isosurface shown at 3σ.

Application of the symmetry expansion workflow [27] allowed us to focus the reconstruction on one of the envelope protein “rafts” containing two asymmetric units. We supposed that the asymmetric reconstruction of a small part of the virion shell would be less affected by the imperfect symmetry of the virions than the reconstruction of the entire viral particle. This approach also allowed us to utilize the local non-uniform refinement algorithm, which is specifically effective for the reconstruction of membrane proteins [28]. By applying a local non-uniform refinement to the symmetry-expanded dataset, we were able to clearly resolve transmembrane domains of both E and M proteins (Figure 3A) and achieved an overall resolution of 3.8 Å (Figure 3B), the same as for the full virion reconstruction. Radial density elongation was also eliminated. This density map was used for the atomic model building. Figure 3C demonstrates the quality of the model fitting into the electron density. The obtained atomic coordinates and density maps were deposited under PDB 8QRH and EMD-18614 respectively.

**Figure 4.**
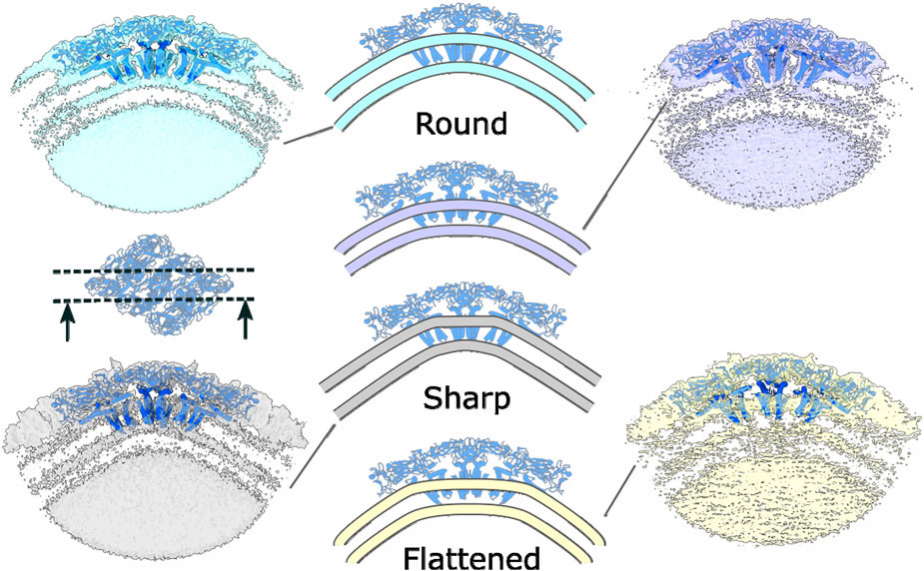
Variability of the single raft conformation, shown on two envelope protein asymmetric units anchoring the membrane. Slice direction is shown with respect to the raft assembly. The unsharpened maps are presented and the isosurface display threshold is adjusted independently to show both the membrane and envelope protein density. The curvature of the membrane varies from smooth to sharp-edged or flattened. The model of a single raft is shown overlapped with the membrane profiles.

We employed a three-dimensional variability analysis [29] and discovered the diversity of the viral membrane curvature and the nucleocapsid density in inactivated virions (Figure 4). The membrane density displays strong variation from almost round to sharp-edged and flattened forms. Both the flattened and sharp-edged features correspond to the asymmetric features identified in the 2D class averages (Figure 1). The membrane curvature variations in orthoflaviviruses occur as a result of the interaction of envelope protein stem-anchor domain with the lipid bilayer [5, 30]. In our case, both E and M protein conformations appear to be the same in all the observed states, as confirmed with the model fitting. This conservation of the form of the transmembrane domains upon a substantial variation of the membrane curvature indicates that their positions may vary relative to the E ectodomain shell. This effect was not observed in the intact TBEV strain Hypr virions [4] or in the virions of other orthoflaviviruses and thus may be caused by the inactivation by formaldehyde.

## Discussion

Atomic resolution structure of the virus particles in the inactivated vaccines has attracted a limited attention from the structural virologists due to their stronger interest to the functioning of the infectious viruses. At the same time, such vaccines are widely used in clinical practice, and detailed understanding of their structure would be important for rationalizing efficiency and further design of advanced formulations. In this study we performed a cryo-EM structure reconstruction of TBEV vaccine strain Sofjin-Chumakov inactivated with formaldehyde.

We observed a very good conservation of the overall virion structure and surface epitopes. The conservation of conformation and location of the E protein domain III is particularly important, because it contains important epitopes recognized by TBEV-neutralizing monoclonal antibodies [31]. We observed a very good conservation of the overall virion structure and surface epitopes. At the same time, transmembrane regions of the envelope proteins, easily observable in the intact virions, could only be visualized by using local refinement.

The imperfect symmetry of the inactivated virions does not allow the use of the conventional single particle workflow for the reconstruction of icosahedral symmetric objects. We attempted to reproduce the processing workflow used for the reconstruction of TBEV strain Hypr virions [4], which involved the full-virion reconstruction with an icosahedral symmetry, and obtained a density map with the overall resolution of 3.8 Å. By comparing the resulting density map with the reconstruction of the intact virion (EMD-3752), we concluded that our reconstruction lacks the density for the transmembrane domains of both E and M proteins (Supplementary Figure S3A). This observation could indicate the presence of structural or conformational heterogeneity in the transmembrane domains. Also, the envelope protein density in our map was slightly distorted, elongated in radial direction (Supplementary Figure S3B). This effect was not present in previously published flavivirus density maps [4, 5, 32–34]. We supposed that both of the observed effects were caused by the ensemble averaging for not perfectly symmetric virion projections. Formaldehyde is a well-known cross-linking agent that attacks the amino group of lysine residues resulting in subsequent covalent binding via the crosslinker. It therefore enhances the rigidity of various structures in the range from protein complexes to whole cells [35]. In comparison, structure of UV-inactivated TBEV strain Kuutsalo-14 [5] did not differ from the native TBEV. Thus, it may be supposed that the envelope protein rafts of the formaldehyde inactivated virions form a quasi-crystalline shell with a lower potential of inter-ectodomain motions compared to the intact virus. This “hull” moves independently from the transmembrane domains and associated lipids, allowing them to displace from their most favorable positions in the intact virions, resulting in the inability to reconstruct the full envelope protein structure using the conventional protocol.

While in the intact virions transmembrane regions are oriented by the ectodomains and nucleocapsids, the inactivation-induced rigidity of the ectodomain hull seems to be more energetically favorable, thus allowing for a disorder of the transmembrane domains. On the other hand, here we clearly demonstrated that antigenic structure of the inactivated virus particle is unchanged, thus proving the integrity and stability of the particles in the TBE vaccine. Our structural data will be useful for the rational design of advanced inactivated vaccines.

## Supplementary Material

TBEV_Cryo_supplement_v5
