# Supplementary material for: Structural diversity of tick-borne encephalitis virus particles in the inactivated vaccine based on strain Sofjin
Source: Emerg Microbes Infect. 2023 Dec 11;13(1):2290833. doi: 10.1080/22221751.2023.2290833 (PMC10930090; doi:10.1080/22221751.2023.2290833)
Supplement: TBEV_Cryo_supplement_v5 [file TEMI_A_2290833_SM2449.pdf]

Supplementary Materials for

Structural Diversity of Tick-Borne Encephalitis Virus Particles in the Inactivated Vaccine Based on Strain Sofjin

Andrey Moiseenko[1], Yichen Zhang[2], Mikhail F Vorovitch[3,4], Alla L Ivanova[3], Zheng Liu[5], Dmitry I Osolodkin[3,4,*], Alexey M Egorov[3,6], Aydar A Ishmukhametov[3,4], Olga S Sokolova[1,2,*]

[1]Faculty of Biology, Lomonosov Moscow State University, Moscow, Russia
[2]Faculty of Biology, Shenzhen MSU-BIT University, Shenzhen, Guangdong, China
[3]FSASI "Chumakov FSC R&D IBP RAS" (Institute of Poliomyelitis), Moscow, Russia
[4]Sechenov First Moscow State Medical University, Moscow, Russia
[5]Kobilka Institute of Innovative Drug Discovery, School of Medicine, Chinese University of Hong Kong, Shenzhen, Guangdong, China
[6]Department of Chemistry, Lomonosov Moscow State University, Moscow, Russia

*** Corresponding authors:** osolodkin_di@chumakovs.su (D.I.O.), sokolova@mail.bio.msu.ru (O.S.S.)

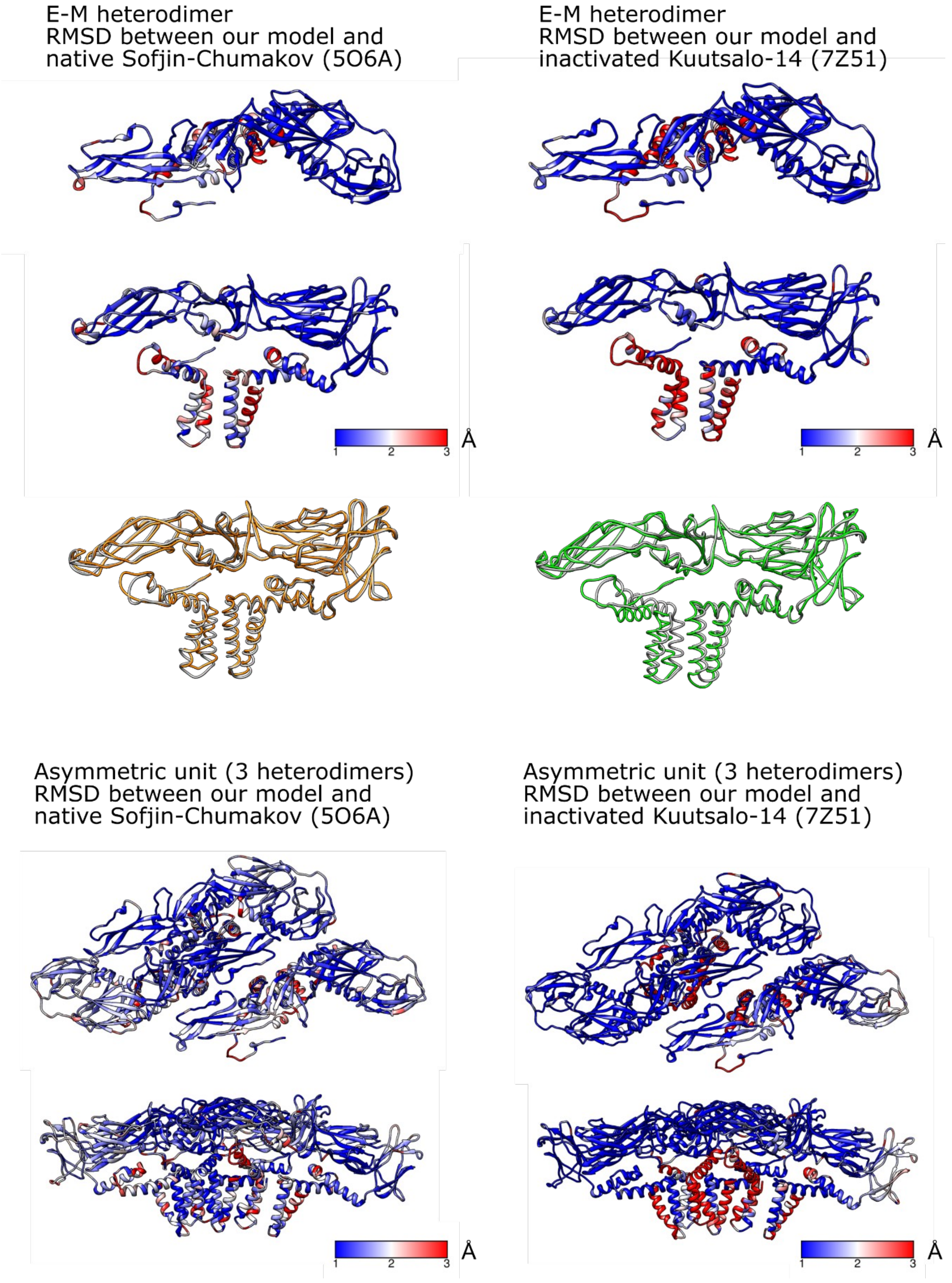

Figure S1: Alignment of E-M protein heterodimer and asymmetric unit. The model built in this study colored by RMSD to the previously published structures of the intact Sofjin-Chumakov and Kuutsalo-14 TBEV strains, PDB codes 5O6A and 7Z51 respectively (Füzik et al., 2018; Pulkkinen et al., 2022). The panel in the middle shows the overlapped view of heterodimers aligned by E protein chain. Our model colored in gray, 5O6A in orange, 7Z51 in green.

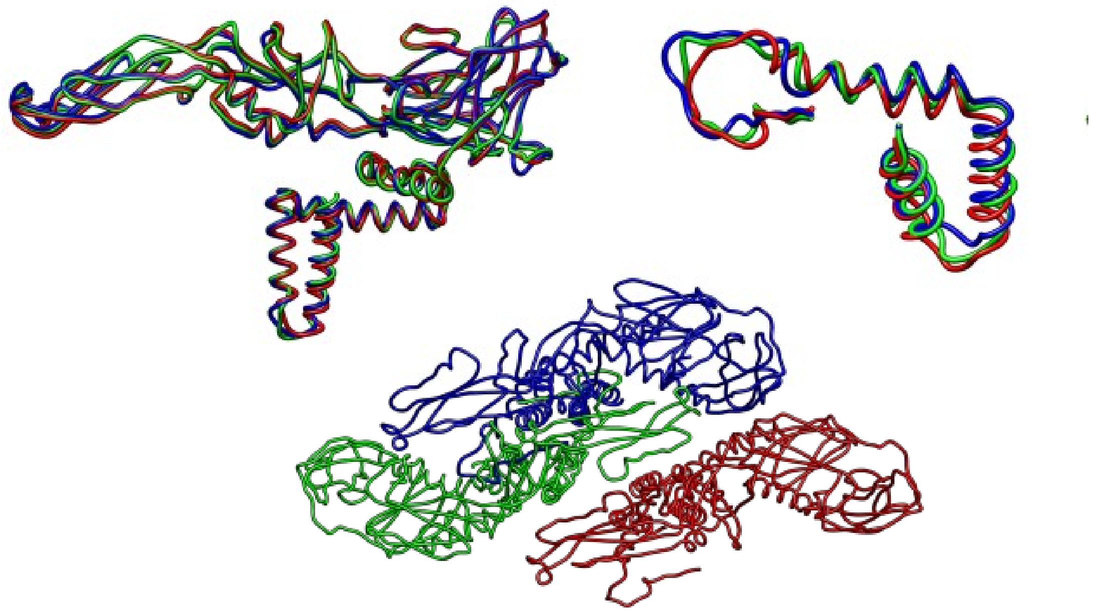

Figure S2: Quasi-equivalence of different E and M protein chains within an asymmetric unit. Three E-M heterodimers from the asymmetric unit model are aligned and shown overlapped to visualize the regions with the most deviations. The heterodimers are color-coded according to their position in the asymmetric unit shown on the bottom panel.

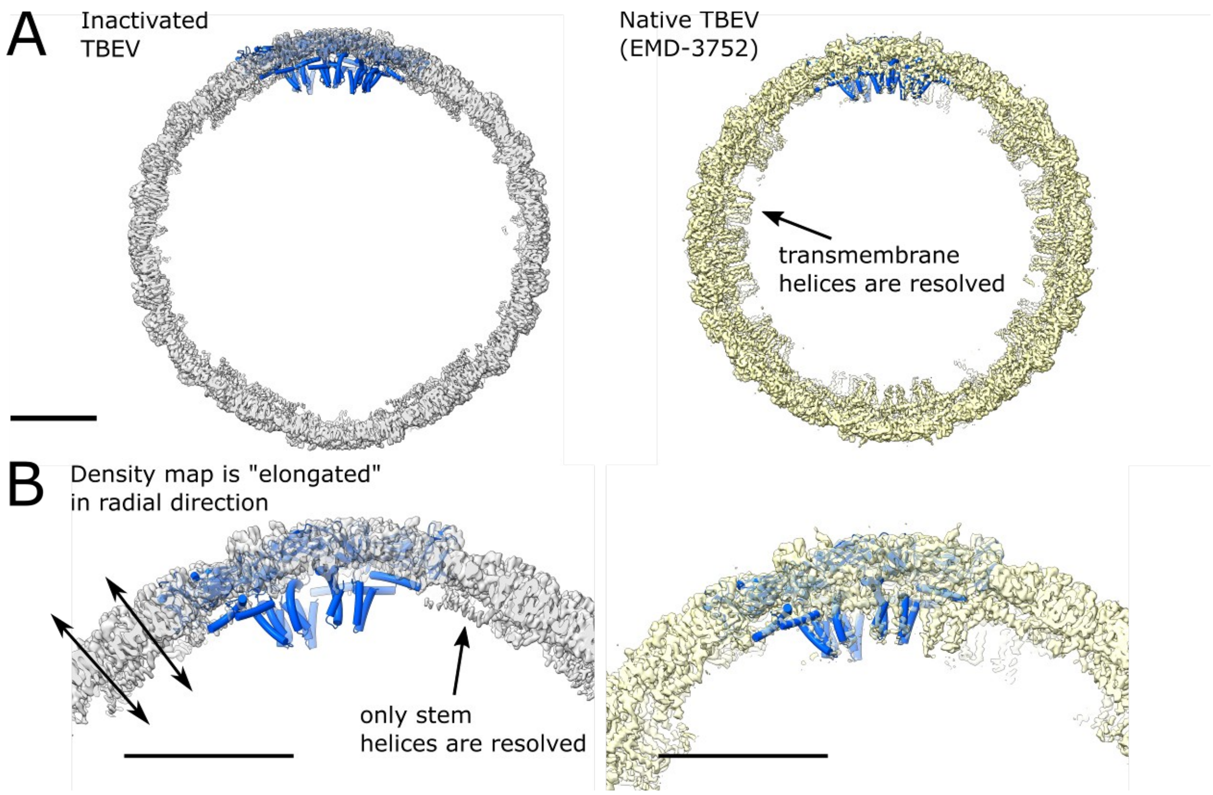

Figure S3: Comparison of the inactivated TBEV density map reconstructed with icosahedral symmetry to the intact virion density map (Füzik et al., 2018). Scalebar 10 nm. (A) Central section through the maps shows that the transmembrane helices are not resolved in our reconstruction. (B) Zoomed views with the fitted asymmetric unit model. The stem helices are clearly visible in both reconstructions, while the transmembrane domains are absent in our reconstruction. Note the radially elongated density. Both effects are supposed to be caused by averaging over ensemble of not perfectly symmetric virion projections. These effects are not present in the intact TBEV reconstruction.

E

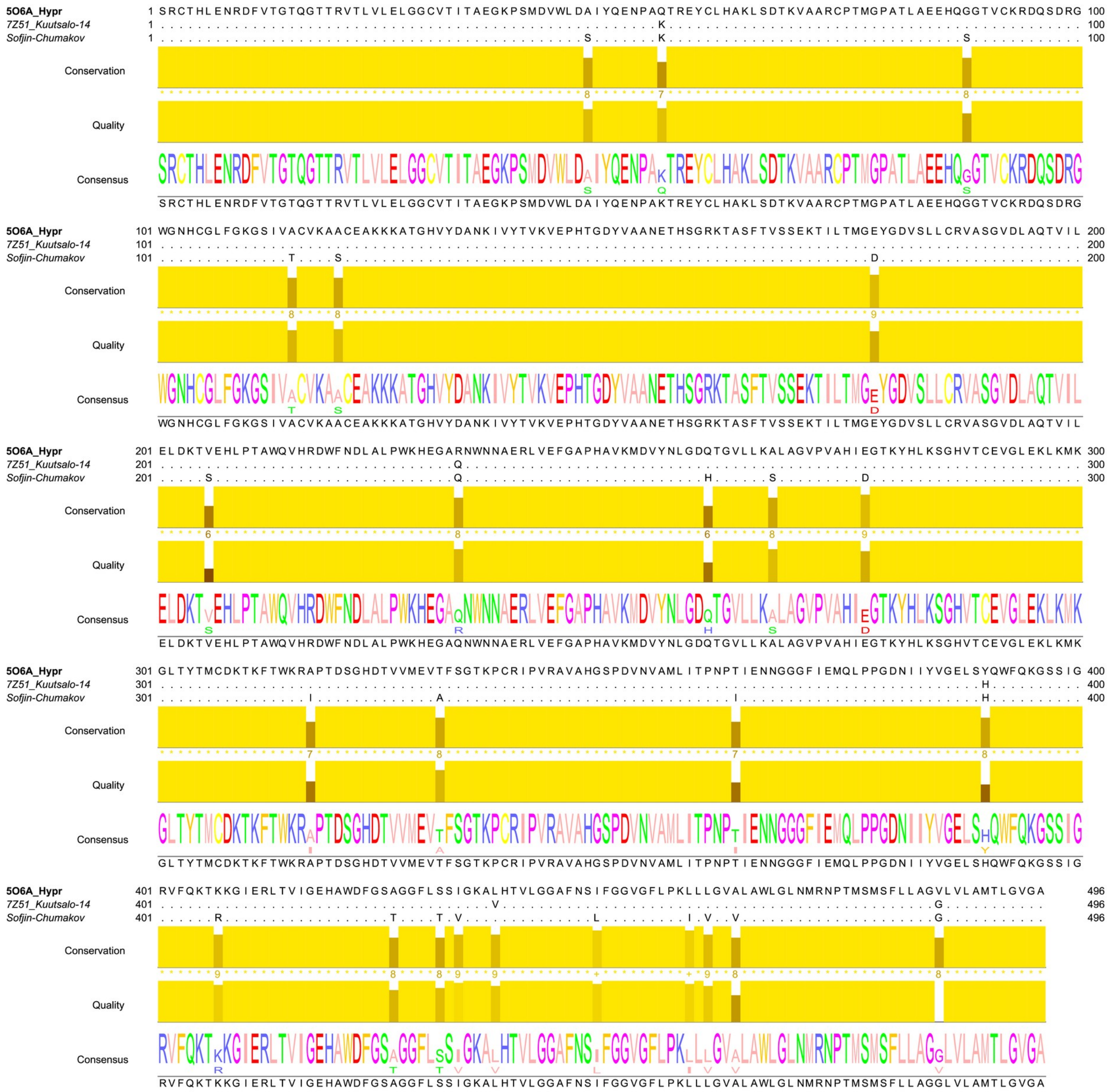

M

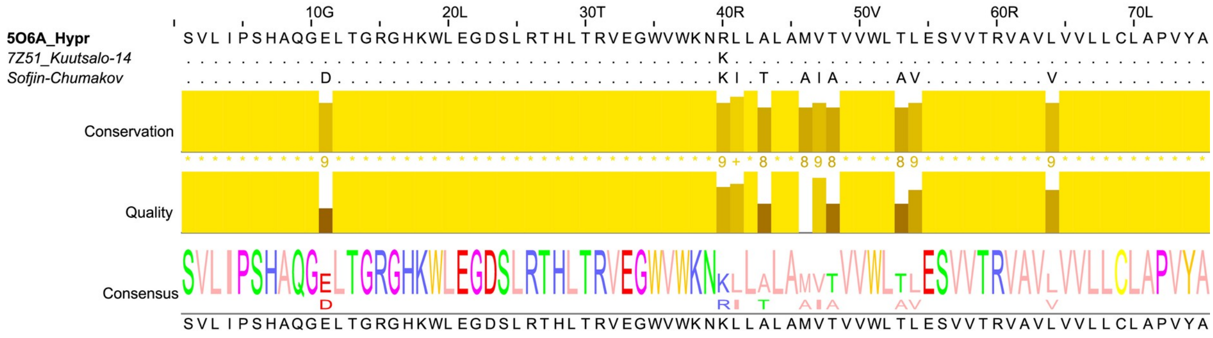

Figure S4: Sequence alignment of E and M proteins from TBEV strains Hypr (PDB ID 5O6A), Kuutsalo-14 (PDB ID 7Z51), and Sofjin-Chumakov. Non-conserved residues with respect to Hypr are shown. The images are rendered with Jalview 2.11.2.7 (10.1093/bioinformatics/btp033).

Supplementary Table 1. Atomic model and density map validation statistics

Reference homology model	PDB: 5O6A
Chains	6
Atoms	12951, no hydrogens
Ligands	NAG x3
Bonds (RMSD)	
Length (Å) (# > 4σ)	0.003 (0)
Angles (°) (# > 4σ)	0.722 (0)
MolProbity score	1.22
Clash score	2.08
Ramachandran plot (%)	
Outliers	0.00
Allowed	3.58
Favored	96.42
Rama-Z (Ramachandran plot Z-score, RMSD)	
whole (N = 1677)	-0.83 (0.19)
helix (N = 411)	-0.18 (0.22)
sheet (N = 486)	-0.53 (0.22)
loop (N = 780)	-0.62 (0.21)
Rotamer outliers (%)	0.00
Cβ outliers (%)	0.00
Cis proline/general	4.8/0.0
Twisted proline/general	0.0/0.0
CaBLAM outliers (%)	2.34
Model vs. Data	
CC (mask)	0.81
CC (box)	0.58
CC (volume)	0.80
Mean CC for ligands	0.79
